# Cell Stress Promotes the Association of Phosphorylated HspB1 with F-Actin

**DOI:** 10.1371/journal.pone.0068978

**Published:** 2013-07-10

**Authors:** Joseph P. Clarke, Karen M. Mearow

**Affiliations:** Division of Biomedical Sciences, Neurosciences Graduate Program, Faculty of Medicine, Memorial University of Newfoundland, St. John’s, Newfoundland, Canada; Center for Cancer Research, National Cancer Institute, United States of America

## Abstract

Previous studies have suggested that the small heat shock protein, HspB1, has a direct influence on the dynamics of cytoskeletal elements, in particular, filamentous actin (F-actin) polymerization. In this study we have assessed the influence of HspB1 phosphorylation on its interaction(s) with F-actin. We first determined the distribution of endogenous non-phosphorylated HspB1, phosphorylated HspB1 and F-actin in neuroendocrine PC12 cells by immunocytochemistry and confocal microscopy. We then investigated a potential direct interaction between HspB1 with F-actin by precipitating F-actin directly with biotinylated phalloidin followed by Western analyses; the reverse immunoprecipitation of HspB1 was also carried out. The phosphorylation influence of HspB1 in this interaction was investigated by using pharmacologic inhibition of p38 MAPK. In control cells, HspB1 interacts with F-actin as a predominantly non-phosphorylated protein, but subsequent to stress there is a redistribution of HspB1 to the cytoskeletal fraction and a significantly increased association of pHspB1 with F-actin. Our data demonstrate HspB1 is found in a complex with F-actin both in phosphorylated and non-phosphorylated forms, with an increased association of pHspB1 with F-actin after heat stress. Overall, our study combines both cellular and biochemical approaches to show cellular localization and direct demonstration of an interaction between endogenous HspB1 and F-actin using methodolgy that specifically isolates F-actin.

## Introduction

The maintenance of cytoskeletal integrity is a key determinant in the survival and growth of cells during stressful cellular episodes, such as radiation, inflammation, or heat stress. In diseases such as the combined motor and sensory neuron disorder Charcot-Marie-Tooth disease, or Alexander disease, perturbations in cytoskeletal integrity can lead to either an establishment or progression of the disease state [1]–[3].

An intrinsic mechanism of importance in modulating cellular responses to stress involves the regulation of a set of highly conserved cellular proteins known as heat shock proteins. These proteins are modulated by cellular stressors and act predominantly as protein chaperones, with some proteins in this family having innate refolding mechanisms to refold damaged proteins [4], [5]. Their cellular function is dependent upon many factors, such as increased protein expression, post-translation modifications, altered oligomerization state and cellular distribution changes. These factors can be further regulated by cell-type specific, stress-activated pathways, including p38 mitogen-activated protein kinase (MAPK) and c-Jun N-terminal kinase pathways [6], [7].

Of particular interest is the small heat shock protein B1 (HspB1) which is a member of the class of small heat shock proteins (sHsps, 15–30 kDa). Like other heat shock proteins, HspB1 acts predominantly as a protein chaperone, but unlike the others, does not display any innate refolding capabilities [8], [9]. HspB1 plays a role in a variety of cellular mechanisms which can be modulated by its posttranslational status [10]–[12]. It is phosphorylated by the stress-activated MAPK pathway involving the activation of MAPK-activated protein kinase-2 (MAPKAP-K2; MK2) by p38-MAPK [13], as well as other kinases that have been implicated in HspB1 phosphorylation [7]. Phosphorylation of multimeric HspB1 on three conserved serines (human Ser15, 78 and 82; rodent Ser15 and 86; hamster Ser15 and 90) by MK2 results in the formation of smaller oligomeric HspB1 structures that are thought to be the main structural units of HspB1 and potentially carry out its main functions [7], [14], [15]. Additionally, changes in the conserved domains of HspB1 can alter the stability of the oligomeric structure of HspB1, and thus regulate its downstream effects on other proteins [11], [16], [17].

HspB1 is involved in the maintenance of cytoskeletal integrity and has been shown to interact with various elements including actin, tubulin, neurofilament, keratins and glial fibrillary acidic protein (reviewed in [18]–[20]). The interactions between HspB1 and actin have been studied extensively in a variety of different experimental systems, and HspB1 has been reported to interact variably with actin filaments or monomeric actin to influence actin polymerization and/or depolymerization [18], [20]–[26]. The necessity of phosphorylation of HspB1 for its interactions with actin has been the subject of much discussion [11], [18], [20], [26], [27]. Models for HspB1 regulation of actin filament dynamics propose that non-phosphorylated monomeric HspB1 inhibits actin polymerization by acting as an actin-capping protein or by binding to and sequestering G-actin monomers, while phosphorylation reverses this effect [18], [21], [28]. However, a direct interation between HspB1 and either G- or F-actin has been questioned [26].

*In vitro* studies have demonstrated interactions between actin and recombinant HspB1 [21], [29]–[32]. Similarly, a number of *in vivo* studies have shown interactions of HspB1 constructs with actin by immunoprecipitating tagged-HspB1 or colocalization at a cellular level of F-actin and HspB1, often employing cells overexpressing various constructs of HspB1 [12], [24], [27], [33]–[35]. We have also used a similar approach in examining the role of HspB1 and its phosphorylation status in contributing to neurite growth from primary sensory neurons [36], [37]. We have observed co-localization of F-actin and HspB1 in neurons, and reported a higher level of localization of F-actin with a non-phosphorylatable HspB1 construct compared to pseudophosphorylated HspB1. In the present study, we were interested in determining whether there is a direct interaction of endogenous HspB1 with F-actin and whether HspB1 phosphorylation would influence this.

In this study we have employed PC12 cells that endogenously express HspB1, and modulated HspB1 phosphorylation status by inhibitor treatment in either a normal or stressed state. Immunocytochemistry, confocal microscopy and western blot analysis were employed to determine the cellular distribution of F-actin and HspB1 following cellular stress. By isolating F-actin specifically or HspB1 via pull-down assays, we investigated any association between the two proteins and the influence of HspB1 phosphorylation status on this association. Our results demonstrate that heat stress induces redistribution of HpsB1 to the cytoskeletal protein fraction and that endogenous HspB1 is associated with F-actin regardless of the phosphorylation status of HspB1. HspB1 is phosphorylated on both serines 15 and 86 after stress and also interacts with F-actin. Immunocytochemistry and confocal imaging show a correspondence with the biochemical analyses, and further suggest a differential compartmentalized distribution of phosphorylated and non-phosphorylated HspB1. Taken together, our data provide new evidence of a direct interaction of HspB1 specifically with F-actin that supports its role in modulating the actin cytoskeletal dynamics.

## Materials and Methods

### Reagents and Antibodies

Standard cell culture, lipofection reagents, streptavidin-coupled Dynabeads® (Cat. No. 112-05D), Dynabeads® Protein A (Cat. No. 100.01D) and Dynabeads® Protein G (Cat. No. 100.03D) were purchased from Invitrogen (Burlington, ON, CAN). *In vitro* actin binding protein assay biochemistry kit (Cat. No. BK013) was obtained from Cytoskeleton (Denver, CO). Biotinylated-phalloidin (Cat. No. P8716) was obtained from Sigma-Aldrich Canada Ltd. (Oakville, ON, CAN). The pharmacologic inhibitor of p38 MAPK, SB203580 (Cat. No. 559389) was purchased from Calbiochem (La Jolla, CA). BCA protein assay solutions, immunoblot reagents and membranes were purchased from ThermoFisher Scientific (Nepean, ON, CAN) and General Electric (Baie D’Urfe, QC, CAN), respectively. Amaxa reagents were purchased from Amaxa Biosystems (Walkersville, MD).

Primary and secondary antibodies used in this study are outlined in Table 1.

**Table 1:** List of primary and secondary antibodies, with experimental dilutions, used for immunoblotting (IB), immunocytochemistry (ICC) and immunoprecipitation (IP).

Antibody	Method	Dilution	Company	Cat
Rabbit anti-actin	IB	1∶500	Sigma-Aldrich	A2066
Rabbit anti-Hsp25	IB/IP	1∶1000	Enzo Life Sciences	SPA-801-F
Rabbit anti-phosphoHsp27(ser15)	IB	1∶1000	Enzo Life Sciences	SPA-525
Rabbit anti-phosphoHsp27(ser82)	IB/ICC	1∶1000	New England Biolabs	2406S
HSP-goat anti-rabbit IgG	IB	1∶10000	ThermoFisher Scientific	31460
Mouse anti-Hsp27	ICC	1∶100	Santa Cruz Biotech	SC-51956
Rabbit anti-phospho-Hsp27 (Ser15)	ICC	1∶100	ThermoFisher Scientific	PA1-018
Alexa Fluor® 488-Phalloidin	ICC	1∶250	Invitrogen	A12379
Alexa Fluor® 405 Goat anti-mouse IgG	ICC	1∶500	Invitrogen	31553
Dylight™ 649 Donkey anti-Rabbit IgG	ICC	1∶500	Jackson Immunoresearch	P711-495-152

### Cell Cultures and Treatments

Neuroendocrine rat pheochromocytoma PC12 cells (ATTC CRL-1721.1; [38]) were grown on collagen coated T-25 culture flasks and maintained at 37°C with 5% CO_2_ in DMEM, supplemented with 10% horse serum, 5% fetal bovine serum and 1% P/S/G. A total of 4×10^6^ cells were used for each sample.

Cell cultures were exposed to 10 µM SB203580 for 1 hr, after which cultures were either incubated for an additional 30 mins, or stressed with heat shock at 42°C for 30 mins (i.e., 1 hr inhibitor treatment → 30 mins heat stress → immediate collection of protein). Optimal inhibitor concentrations were determined empirically [39]. Immediately after treatments, cells were collected and then lysed with actin stabilization buffer (ASB: 1% Triton-X 100, 0.1% sodium dodecyl sulfate (SDS), 10 mM ethylenediaminetetraacetic acid, 1% sodium deoxycholate, 200 pM sodium vanadate, 200 pM sodium fluoride, 1 complete protease inhibitor cocktail tablet, 0.5 mM ATP and Tris-Buffered Saline, pH 7.4). After lysis, samples were either centrifuged at 15,000×g for 10 min to be separated into Triton X-100 soluble (cytosolic; lysate) and Triton X-100 insoluble (cytoskeletal; pellet) samples, or were left as total lysate (crude sample).

### In vitro F/G-Actin and Biotinylated-Phalloidin Pull-down

Non-muscle actin was prepared *in vitro* into globular actin (G-Actin) or filamentous actin (F-actin), as per the manufacturer’s protocol (Cytoskeleton), and then subsequently pulled-down with 5 µg of biotinylated-phalloidin. Briefly, 2.5 µg and 5.0 µg samples of both G-Actin and F-actin were diluted in a total volume of 200 µL in TBS, and 5 µg of biotinylated-phalloidin was added to each sample. G-actin/biotinylated-phalloidin and untreated samples (actin-containing sample lacking phalloidin) were incubated for one hour at 4°C, with constant rotation on a bench-top rotator. F-actin/biotinylated-phalloidin and untreated samples (actin-containing sample lacking phalloidin) were incubated for one hour at room temperature (∼22°C), with constant rotation on a bench-top rotator. After one hour, 20 µL of streptavidin-coupled Dynabeads® were added only to the biotinylated-phalloidin treated samples and these were also incubated for one hour at 4°C (G-Actin sample), or room temperature (F-actin sample), with constant rotation on a bench-top rotator. Pull-down at this point was considered to be complete, the samples were then isolated using a block magnet and 20 µl of 2× loading dye was added. Supernatants from pull-downs were collected and used for subsequent biochemical analysis to assess efficiency of the pull-down. Dynabead® pellets were washed three times with TBS, with the samples being used for biochemical analysis after the third wash. Immunoblots of pull-downs (phalloidin interacting samples), untreated supernatants (no biotinylated-phalloidin or Dynabeads® added) and supernatants after pull-down (non-phalloidin interacting samples) were then carried out. More specifically, 65×µL of untreated sample and supernatants after pull-down (approximately 1/3 of original volumes), were electrophoresed along with the precipitated samples and immunoblotted with anti-actin antibodies.

### Pull-Down Assay – cellular samples

Previous studies have shown that biotinylated-phalloidin can be used to specifically pull-down F-actin from tissue [40]. By adapting this technique to our study, we performed pull-downs on Triton X-100 insoluble (cytoskeletal) protein samples resuspended in ASB, and crude total protein samples using biotinylated-phalloidin. Both samples were used in order to assess F-actin pull-down efficiency, which was ultimately determined to be similar. Biotinylated-phalloidin (5×µg) was added to the protein samples and they were incubated for one hr at 4°C with constant rotation. Subsequently, 20×µL of streptavidin-coupled magnetic Dynabeads® were added and this was incubated for an additional hour at 4°C with constant rotation. The magnetic beads were isolated using a block magnet and washed three times with PBS, with the samples being used for biochemical analysis after the third wash. Pull-downs of HspB1 were performed similarly, using rabbit anti-HspB1 antibody (5×µg) and a 1∶1 mixture of Dynabeads® A/G that were substituted for biotinylated-phalloidin and streptavidin-coupled magnetic Dynabeads®, respectively.

### Biochemical Analysis of Protein Fractions and Pull-Downs

Protein concentrations of lysates were determined using the BCA protein assay. Equivalent amounts of protein (20–25×µg) were electrophoresed on a 10% SDS-PAGE, separated, and transferred onto a nitrocellulose membrane; Ponceau Red staining was used to assess the equivalency of protein loading. Blots were then blocked either with 3% skim milk, or 5% BSA solution, depending on whether phospho-antibodies were used or not, incubated at 4°C overnight with primary antibodies (Table 1) and developed the next day. Bands were detected using peroxidase conjugated secondary antibodies and SuperSignal® West Pico Chemiluminescent Substrate.

Analysis of the pull-down samples was carried out as follows. After the final wash of the pull-down pellets, the supernatants were discarded and loading dye with dithiothreitol (DTT) was added. These samples were pulse sonicated (3 pulses of 3 sec each with the power set to 35% of maximum using a model 16–850 Virsonic Cell Disrupter) and then separated once again using a block magnet. The supernatants were loaded onto a 10% SDS-PAGE and treated as outlined above.

### Immunocytochemistry of PC12 Cells

PC12 cells were cultured on collagen-coated 16-well chamber slides at ∼1000 cells/well. Cells were treated with 10 µM SB203580 (p38 MAPK inhibitor), with and without a heat stress at 42°C for 30 mins (1 hr inhibitor treatment → 30 mins heat stress → immediate fixation of cells). After treatment, cells were fixed immediately using 4% paraformaldehyde in PBS for 20 mins, permeabilized in 0.1% Triton X-100, and blocked with 10% horse serum in PBS. Cells were incubated with primary antibodies at appropriate concentrations (Table 1) for 16–20 hrs at 4°C, rinsed with TBS supplemented with 0.025% Tween 20, pH 7.2 (TBST), and subsequently incubated with fluorophore-conjugated secondary antibodies at appropriate concentrations (Table 1) for 1–2 hr. Cells were washed again three times with TBST and cover-slipped with Gelvatol™. Images were acquired in each of three channels (405 nm, 488 nm and 649 nm) by laser scanning confocal microscopy with sequential Z-stage scanning (Olympus Flowview 1000 confocal laser scanning microscope). Image acquisition for the different samples was carried out using similar scanning parameters (laser power, HV PMTs, and number of optical slices). Scanned stacks were compiled as individual images, and composite digital images were prepared in Adobe Photoshop CS.

### 3-Dimensional Imaging of Cellular Expression

Immunocytochemical confocal images were volume rendered in 3 dimensions (3D) using Imaris software (Bitplane Inc., South Windsor, CT). Confocal image stacks were initially saved as.oib files and then imported into Imaris. Images were cropped in 3D to focus on a region of interest and rendered employing the Surfaces tool within the Surpass module of Imaris. Images were then saved as.tif files and Adobe Photoshop CS was used to prepare composite images.

### Densitometric and Statistical Analysis

Densitometric analyses of triplicate Western immunoblots were carried out using ImageJ and a calibrated grey value scale (NIH, Bethesda, Maryland); ratios of HspB1 expression (total and phosphorylated) were compared to actin expression. Densitometric analyses of confocal images (using average gray scale assessment) were also carried out using ImageJ, with average grey value expression of pSer15-HspB1, pSer86-HspB1 and total HspB1 being calculated. Statistical analyses were performed using GraphPad Prism 4 (GraphPad Software, San Diego, CA) with significance determined by one-way ANOVA and post hoc testing via Tukey’s test.

## Results

### Cellular Distribution of Total HspB1 and Phosphorylated HspB1

We initially examined the cellular distribution of HspB1, phospho-HspB1 and F-actin in control conditions compared to heat stress. PC12 cells were either not treated (vehicle control) or treated with the p38 MAPK inhibitor SB203580 (10 µM) for 1 hr prior to stress; the cells were then heat-shocked for 30 min at 42°C, and fixed immediately. The distribution of HspB1, phospho-HspB1 and F-actin was visualized by confocal imaging. Cellular localization of HspB1 was determined with immunocytochemistry by using antibodies specific for pSer15-HspB1, pSer86-HspB1 and total HspB1, while F-actin was specifically visualized with fluorescently labeled Phalloidin.

**Figure 1:**
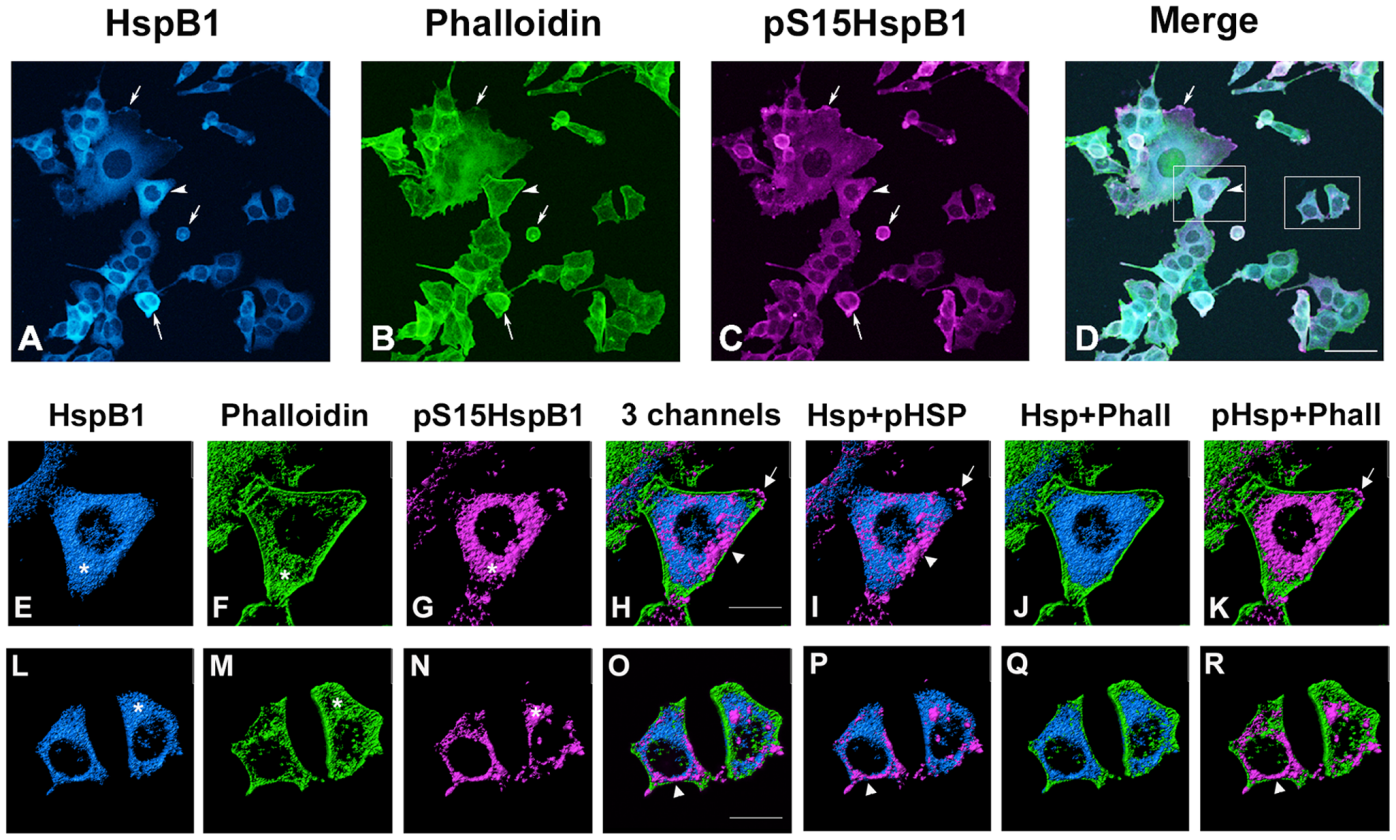
Cellular localization of HspB1 and F-actin in control PC12 cells. Low passage PC12 cells were cultured on collagen-coated coverslips, and processed for immunocytochemistry using antibodies directed against total HspB1 (Stressgen SPA801) or phospho-S15-HspB1 followed by fluorescently tagged secondary antibodies; F-actin was labelled using Alexa-488-phalloidin. Panel A-D: PC12 cells visualized with confocal microscopy; images represent the z-stack projections. A – HspB1 (blue); B- phalloidin (green); C-pS15-HspB1 (magenta); D- merged image. Panels E-K, L-R: three-dimensional rendering of immunostained surfaces as compiled by Imaris software of cells (boxes in D) selected from the low power view. E, L -HspB1 (blue); F, M - Phalloidin (green); G, N - pHspB1(magenta); H, O - all 3 together; I, P - HspB1+pHspB1; J, Q – HspB1+Phalloidin; K, R - pHspB1+Phalloidin. Scale bar –50 µm, D; 10 µm, lower panels.

In the control conditions, HspB1 was localized primarily throughout the cytosol, in a somewhat granular appearance. In some cells with lamellopodia, HspB1 was observed at the leading edges (Fig. 1A, arrows). pHspB1 was also detected throughout the cells, and at the leading edges of lamellopodia or ruffles (Fig. 1C arrows). ICC of pHspB1-Ser15 is presented, although the pattern of pHspB1-Ser86 staining is similar (data not shown). F-actin (as detected by Alexa-488 Phalloidin labelling) was observed at the cellular periphery outlining the cell boundaries (Fig. 1B, arrowhead), as well as being found in a more punctate distribution throughout the cell. There was co-localization observed between F-actin and HspB1, and to a certain extent with the pHspB1 (Fig. 1D).

**Figure 2:**
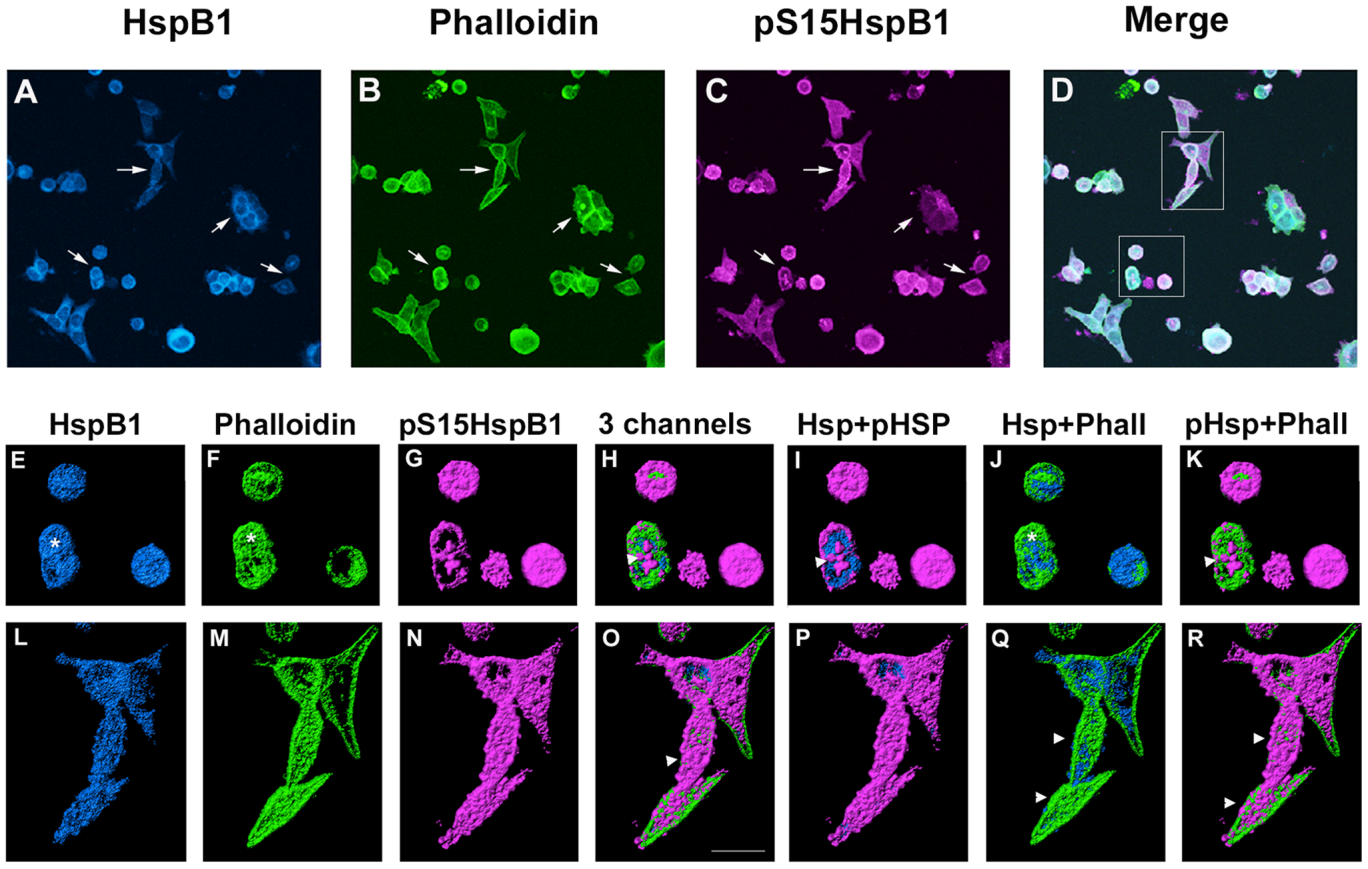
Cellular localization of HspB1 and F-actin in PC12 cells subsequent to a heat shock. Low passage PC12 cells were cultured on collagen-coated coverslips and were subjected to a 30 min heat shock (HS) followed by immediate fixation, and processing for immunocytochemistry using antibodies directed against total HspB1 or phospho-S15-HspB1 followed by fluorescently tagged secondary antibodies; F-actin was labelled using Alexa-488-phalloidin. Panel A-D: PC12 cells visualized with confocal microscopy; images represent the z-stack projections. A – HspB1 (blue); B – phalloidin (green); C-pS15-HspB1 (magenta); D– merged image. Panels E–K, L–R: three-dimensional rendering of immunostained surfaces as compiled by Imaris software of cells (boxes in D) selected from the low power view. E, L -HspB1 (blue); F, M - Phalloidin (green); G, N – pHspB1(magenta); H, O – all 3 together; I, P - HspB1+pHspB1; J, Q – HspB1+Phalloidin; K, R – pHspB1+Phalloidin. Scale bar –10 µm.

The HS treatment resulted in the rounding of the cells and the collapse of the cytoskeleton and redistribution of HspB1 to a peri-nuclear location (Fig. 2A), a classical reaction of many cells to heat shock. Ruffles and membrane blebbing were observed on the surface of many of the cells (Fig. 2A–D). In these cells, there was again a distinct colocalization of the HspB1, pHspB1 and actin in a punctate or granular pattern within the cells, and associated with the membrane (Fig. 2A–C, arrows). There was an apparent increase in the amount of pHspB1 staining and this appeared to be colocalized with the F-actin (Fig. 2B–C, arrows).

**Figure 3:**
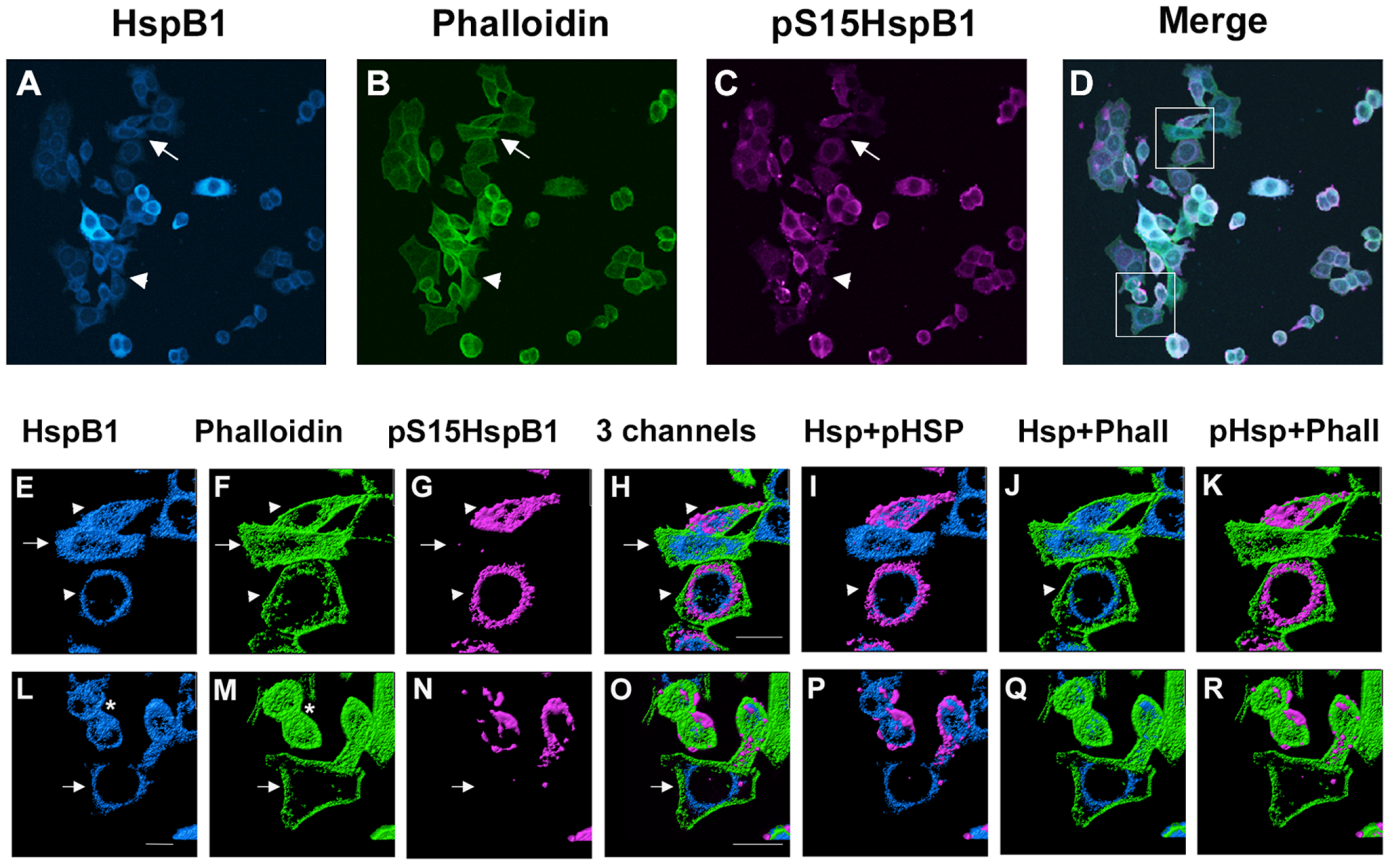
Cellular localization of HspB1 and F-actin in PC12 cells subsequent to a heat shock in the presence of a p38MAPK inhibitor. Low passage PC12 cells were cultured on collagen-coated coverslips, incubated with 10 µM SB203580 for 1 hr prior to and during a 30 min heat shock (HS), followed by immediate fixation and processing for immunocytochemistry using antibodies directed against total HspB1 (Stressgen SPA801) or phospho-S15-HspB1 followed by fluorescently tagged secondary antibodies; F-actin was labelled using Alexa-488-phalloidin. Panel A–D: PC12 cells visualized with confocal microscopy; images represent the z-stack projections. A – HspB1 (blue); B- phalloidin (green); C-pS15-HspB1 (magenta); D- merged image. Panels E–K, L–R: three-dimensional rendering of immunostained surfaces as compiled by Imaris software of cells (boxes in D) selected from the low power view. E, L -HspB1 (blue); F, M - Phalloidin (green); G, N – pHspB1(magenta); H, O – all 3 together; I, P – HspB1+pHspB1; J, Q – HspB1+Phalloidin; K, R - pHspB1+Phalloidin. Scale bar –10 µm.

In the presence of the p38MAPK inhibitor (SB) plus HS (SB+HS), the pHspB1 staining was somewhat attenuated (Fig. 3C), appearing similar to the control condition. Many of the cells were rounded and the F-actin staining appear less distint than in the HS alone condition (Fig. 3B compared to Fig. 2B). There was still colocalization of the HspB1 and F-actin, but closer examination of the cells revealed that there seemed to be an absence of the blebbing/ruffling that was apparent with HS alone; the staining of HspB1 and F-actin did not appear granular or punctate in nature (compare Fig. 2A to 3A, 2B to 3B).

Three-dimensional images of each channel separately and in combination provide a more informative view of the distribution of HspB1 and actin under each of the conditions. Representative cells (or groups of cells) were selected from the each of the images presented in Fig. 1–3 and 3-dimensional surfaces for each channel rendered using Imaris software. Confocal image stacks were obtained with sequential z-stage scanning over a 5–10 µm top-bottom distance of the immunostained cultures, image stacks imported into Imaris software (Bitplane Corp), and 3D surfaces and cellular volumes displayed.

In cells selected from the control condition (Fig. 1E–R), HspB1 is distributed rather evenly throughout the cytoplasm, and colocalized with both F-actin and basal levels of pHspB1 for the most part. However, pHspB1 is also clearly observed at the leading edges of the cells (perhaps in focal contacts/adhesions) and co-localized with F-actin in these areas (Fig. 1J, Q). F-actin is associated with cortical areas (particularly noticeable in Fig. 1F, J, K), as well as being found distributed throughout in apparent colocalization with HspB1 and pHspB1. Note that the cortical F-actin does not appear to completely overlap with HspB1 in these examples (Fig. 1E, F, H, J).

With HS (Fig. 2E–R), many of the cells have rounded up, and in these cells both HspB1 and F-actin appear condensed and collapsed (Fig. 2E–K). There is an increased amount of pHspB1 and this appears associated mainly with the surfaces of the cells (Fig. 2G–I). In the second series of cells (Fig. 2 L–R), the blebbing noted above is more clearly observed showing that pHspB1 and F-actin apparently associated with the surface of cell.

In cells subjected to HS in the presence of the inhibitor (Fig. 3 E–R), the phosphorylation of HspB1 is attenuated, but what little is present is still associated with actin and membrane blebbing. In one cell, where the HspB1 has become mainly perinuclear, much of the F-actin remains peripheral, although there is still some overlap with the perinuclear HspB1. In these cells, even where this is essentially no pHspB1 detectable, HspB1 and F-actin still colocalize.

Interestingly, in cells where there is no detectable pHspB1 (arrowheads, Fig. 3N–O), F-actin appears to be retained in a cortical organization though not associated with HspB1. In another example (Fig. 3F–G), in the absence of pHspB1, there is still colocalization with HspB1 and a more cellular distribution of the F-actin staining, perhaps indicative of the presence of smaller actin filaments.

These results show colocalization of HspB1 and F-actin in both control and stressed conditions, and suggest that HspB1 does not necessarily require phosphorylation in order to interact with F-actin.

Statistical analysis of the imaging data (described in the Methods) confirmed the qualitative expression differences, showing pHspB1 was significantly upregulated with stress and this upregulation attenuated by the inclusion of SB203580; there was no significant difference in the expression of total HspB1 in any condition (Figure S1).

### Biochemical Assessment of Hsp and F-actin Distribution with Stress

To further investigate whether the immunocytochemical colocalization represents a molecular association of HspB1 with F-actin, we carried out a series of biochemical assays using analyses of protein lysates from cells treated as noted above (control, 30 min HS, SB, and SB+HS). Cells were lysed immediately at the end of the experimental treatment (no recovery from HS) and the intracellular distribution of HspB1 and actin was determined by Western blotting of 3 different preparations: the Triton X-100 soluble (cytosolic lysate), Triton X-100 insoluble (cytoskeletal pellet) and unfractionated total cellular lysate.

**Figure 4:**
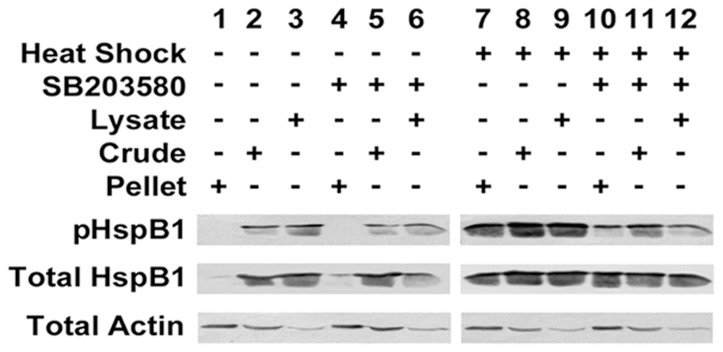
HspB1 is phosphorylated and redistributed to the cytoskeletal fraction during cellular stress. PC12 cells were treated as described in the Methods, subsequently lysed and separated by cellular fractionation into Triton X-100 soluble (cytosolic; lysate) and Triton X-100 insoluble (cytoskeletal; pellet) samples, or were left as crude total protein samples. Untreated cells (Lanes 1–3); treated with 10 µM SB203580 (Lanes 4–6); heat shocked cells (Lanes 7–9); cells treated with inhibitor and heat shock (Lanes 10–12). Western blots were sequentially probed with antibodies to pS15-HspB1, pS86-HspB1, HspB1 and actin; blots were stripped after each probe and prior to the next. Note the increased amount of HspB1 in the cytoskeletal fraction after HS, as well as the increased amount of phosphorylated HspB1.

Under control conditions, total HspB1 was primarily found in the cytosolic and crude total protein fractions, and there was also a low level of pHspB1 detected in these samples (Fig. 4). After heat shock (HS), there was an increase in HspB1 in the cytoskeletal fraction (compare lane 1 with lane 7) associated with increased phosphorylation of HspB1. With the addition of the p38 MAPK inhibitor SB203580, HspB1 phosphorylation was attenuated as expected, but there appeared to be little effect on the redistribution of the HspB1 to the cytoskeletal fraction (Fig. 4). While HS does result in increased expression of HspB1 over a longer time frame (>3 hrs recovery after HS [41]), in these experiments cells were sampled immediately after the termination of the HS treatment to assess early effects of HspB1 phosphorylation.

Actin was observed in all cell fractions with an increased expression in the cytoskeletal fraction (Fig. 4, eg., lanes 1 vs 3), but with little detectable difference in distribution with stress. Since the antibodies used to probe for actin detect both filamentous and globular actin, one cannot distinguish between the two species by Western blotting. However, it is generally accepted that the actin associated with the cytoskeletal fraction represents F-actin, while the cytosolic fraction represents globular actin [42].

### Determining the Interaction(s) of HspB1 and F-actin

#### Pull-Down and Immunoprecipitation assays

While HspB1 is reported to interact with actin, there has been little evidence of an interaction with F-actin specifically in a cellular model (in the absence of overexpression of tagged HspB1). We thus investigated whether there was an association of HspB1 with F-actin, and if so, whether the phosphorylation state of HspB1 influenced this interaction.

**Figure 5:**
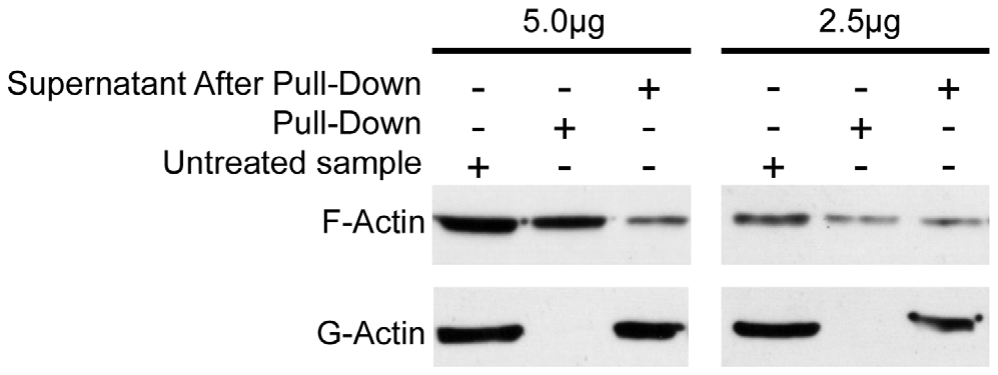
Specificity of the Phalloidin pull-down for F-actin. *In vitro* preparations (2.5 or 5 µg) of globular actin (G-actin) and filamentous actin (F-actin) were prepared using an *in vitro* actin–binding assay kit (Cytoskeleton) and incubated with 5 µg of biotinylated-phalloidin as detailed in the Methods section. The resulting solutions were separated into supernatant (non-phalloidin interacting) and pull-down (phalloidin interacting) samples and assayed by Western blotting. Additionally, untreated G- or F-actin samples were also probed. Note that biotinylated-phalloidin selectively precipitates F-actin, but not G-actin, and that this pull-down is reasonably efficient.

To specifically isolate F-actin we employed a biotinylated phalloidin pull down protocol. The efficacy and specificity of this approach was first assessed using solutions of polymerized F-actin and G-actin. As shown in Fig. 5, the biotinylated phalloidin specifically pulled down F-actin (top panels), with no G-actin being observed in the precipitated samples (bottom panels).

Experimental samples were incubated with biotinylated-phalloidin followed by precipitation of the captured complexes with streptavidin-linked magnetic beads. Precipitated fractions were then subjected to SDS-PAGE and immunoblotted to detect pHspB1, total HspB1 and actin. Although we were primarily interested in the F-actin and HspB1 in the cytoskeletal fractions, we initially performed the experiments on both the total cell lysate (N = 2 experiments) and the cytoskeletal fraction (N = 3 experiments).

**Figure 6:**
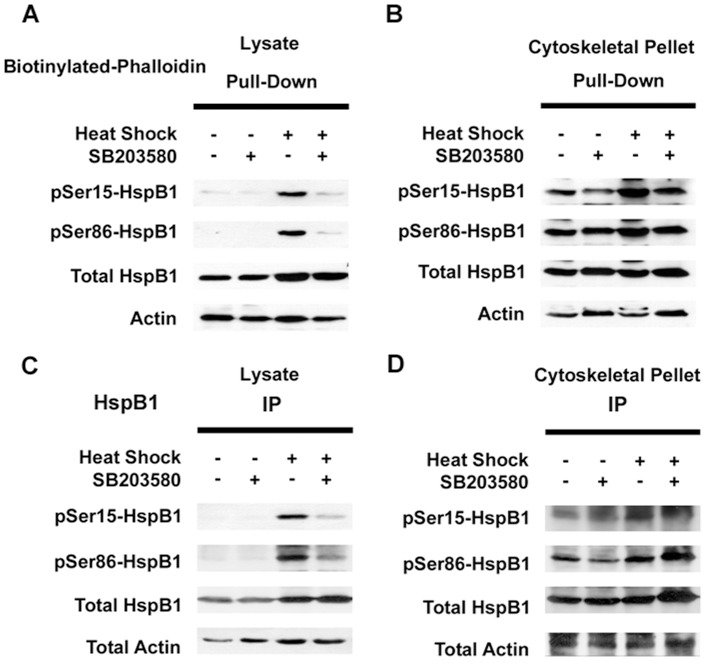
HspB1 is associated with F-actin. Representative blots showing precipitation of F-actin complexes (with biotinylated phalloidin, A–B) and IPs of HspB1 (C–D) from total cellular lysates (A, C) or the cytoskeletal pellet fraction (B, D). PC12 cells were treated as described in the Methods. Cell cultures were exposed to 10 µM SB203580 for 1 hr, after which cultures were either incubated for an additional 30 mins, or stressed with heat shock at 42°C for 30 mins; control cells were not treated. Immediately after treatments, cells were collected, lysed with actin stabilization buffer; samples were separated for analysis as total cell lysate or further fractionated into the TritonX-100 insoluble cytoskeletal pellet. Samples were incubated with biotinylated-phalloidin followed by precipitation of the captured complexes with streptavidin-linked magnetic beads. Precipitated fractions were then subjected to SDS-PAGE and sequentially immunoblotted to detect pHspB1, total HspB1 and actin. Pulldown of F-actin also captures HspB1 in both the cell lysates (A) and the cytoskeletal fraction (B); similarly the HspB1 IPs also bring down actin and this is enhanced in the cytoskeletal fraction.

**Figure 7:**
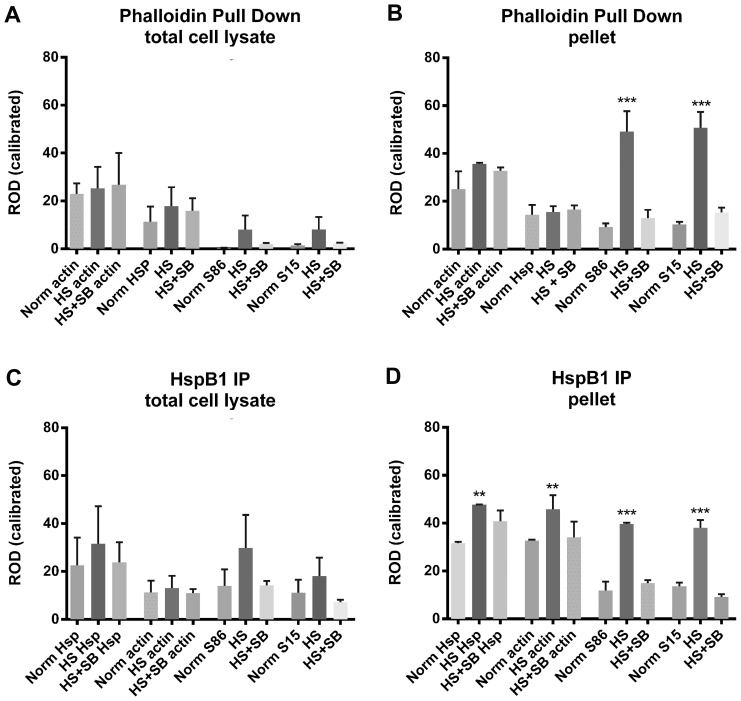
Densitometric analyses of the amounts of F-actin and HspB1 in the precipitated complexes. Western blots were scanned and densitometric analysis of bands carried out using Image J and a calibrated gray scale standard. The bars are the mean ROD (±SEM) of paired values for Actin, HspB1 (Hsp), pS86-HspB1 (S86) and pS15-HspB1 (S15) from each of 3 separate experiments for the cytoskeletal fraction samples (pellet) and from 2 separate experiments for total cell lysates. Panel A – Phalloidin pulldown from total cell lysates; B – Phalloidin pulldown from the cytoskeletal fraction; C – HspB1 IP from total cell lysates; D – HspB1 IP from the cytoskeletal fraction. Norm – control; HS – Heat Shock; HS+SB – Heat shock+SB203580. ** p<0.01; *** p<0.001.

The Western blots of representative phalloidin pull-down experiments are presented in Fig. 6 (panels A, B) while Fig. 7 presents the graphical analyses of these experiments from the total cell lysate (Fig. 7A) and the cytoskeletal fraction (Fig. 7B). As shown in Fig. 6A, pulldown of F-actin from the total cellular lysate captured HspB1 in the control situations and this was increased with HS. In the control situations, little of the HspB1 associated with the F-actin was phosphorylated. However, after HS, the fraction of pHspB1 associated with F-actin increased, and this was inhibited in the presence of SB203580.

Phalloidin pulldown from the the TritonX- insoluble (cytoskeletal pellet) fraction, which should be enriched in F-actin, is shown in Fig. 6B. HspB1 was precipitated with the F-actin, although there were no significant differences between the untreated and HS conditions for either the F-actin or HspB1 (Fig. 7B). In contrast to the total cellular lysate, much of the HspB1 found in the complexes with F-actin was phosphorylated and was significantly increased with HS, and blocked with the inhibitor (Fig. 7B).

In order to provide some measure of specificity, since it was possible that large complexes in the cytoskeletal precipitates could be non-selectively ‘trapping’ HspB1, we carried out immunoprecipitation of HspB1 under the same conditions as noted above. The results of representative HspB1 IP experiments are presented in Fig. 6 C–D, with graphical analyses presented in Fig. 7 C–D. HspB1 was effectively immunoprecipitated from the cell lysates, and there was some increase after HS (Fig. 6C). In the control conditions little of the HspB1 was phosphorylated (similar to what was observed for the F-actin pulldown), with increases in pHspB1 observed after HS and blocked in the presence of the inhibitor. Here we also observed actin being co-precipitated with the HspB1 (Fig. 6C). In the cytoskeletal fraction, as with the F-actin-specific pull-downs, IP of HspB1 showed that with HS there was a significant increase in the amount of HspB1 precipitated (Fig. 7D). This indicated a redistribution from the cytosol to the cytoskeletal fraction and concomitantly, also a significant increase in the amount of actin co-precipitating with HspB1 (Fig. 7D). pHspB1 was significantly increased after HS and this increase was attenuated by treatment with the inhibitor (Fig. 7D).

**Figure 8:**
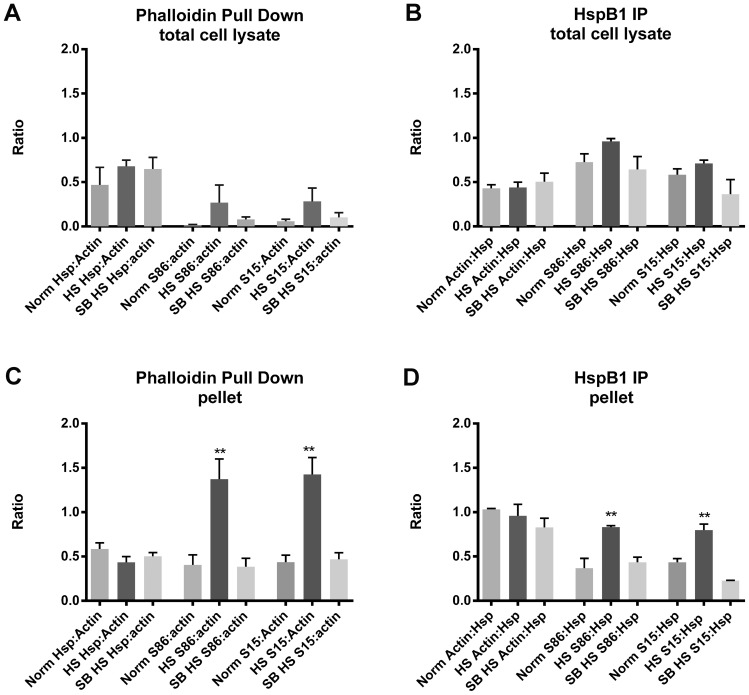
Changes in the relative amounts of the association of F-actin and HspB1. Densitometric data of F-actin and HspB1 expression in pulldowns presented as ratios of HspB1:F-actin or Actin:HspB1, as well as the pHspB1:F-actin or pHspB1:HspB1. Panel A - ratios for Phalloidin pulldown from total cell lysate; B - ratios for HspB1 IP from total cell lysate; C - ratios for Phalloidin pulldown from cytoskeletal fraction (pellet); D – ratios for HspB1 from the cytoskeletal fraction. ** p<0.01.

The ratios of HspB1 and pHspB1 relative to F-actin are presented graphically in Fig. 8 (Fig. 8A,C for F-actin; Fig. 8B,D for HspB1). With respect to the F-actin pulldown, the ratio of HspB1:actin does not differ between the total cell lysate and the pellet, however it is clear that HS significantly increases the fraction of phosphorylated HspB1 precipitated with F-actin (Fig. 8C). Although the ratio of actin:HspB1 differs between the cell lysates and the pellet, with more actin being associated with HspB1 in the cytoskeletal fraction, again there is a significant increase in the phosphorylated fraction of HspB1 with HS.

The results of the biotinylated pulldowns suggest that not only does HspB1 directly interact with F-actin, but also that a relatively large fraction of this HspB1 is phosphorylated in stressed cells. The HspB1 IP experiments also point to an interaction with F-actin selectively (results from the cytoskeletal fraction), although we cannot rule out an interaction of HspB1 also with non-filamentous actin (results from the total cell lysates, where both F- and G-actin should be present).

## Discussion

In this study, we investigated the interacton of HspB1 with cytoskeletal F-actin and the influence of phosphorylation on this association. We demonstrate cellular colocalization of HspB1 and F-actin, as well as a molecular interaction between endogenous HspB1 and F-actin under both control and stressed conditions. Our pulldown and IP results indicate that HspB1 and F-actin are normally associated to some degree, but cell stress results in an enhanced interaction especially of pHspB1. Overall, our data provide support for a direct association of HspB1 with F-actin.

### HspB1 Interactions with Actin

HspB1 has been reported to interact variably with actin filaments or monomeric actin to influence actin polymerization and/or depolymerization [18], [20]–[26]. *In vitro* studies have demonstrated interactions between actin and recombinant HspB1 [21], [29]–[32]. Similarly, a number of *in vivo* studies have shown interactions of HspB1 constructs with actin by immunoprecipitating tagged-HspB1, or colocalization at a cellular level of F-actin and HspB1, often employing cells overexpressing various constructs of HspB1 [12], [24], [27], [33], [34]. However, a direct interaction between HspB1 and either G- or F-actin has been questioned [26], [43].

While there have been numerous studies describing an interaction between F-actin and Hspb1 using purified solutions of each, there has been much less evidence of such interactions in a cellular system. Models for HspB1 regulation of actin filament dynamics propose that non-phosphorylated monomeric HspB1 inhibits actin polymerization by acting as an actin-capping protein or by binding to and sequestering G-actin monomers, while phosphorylation reverse this effect and allows for polymerization or filament elongation [18], [21].

### Role of HspB1 and Actin Interactions in Unstressed Cells

HspB1 interactions with actin and other cytoskeletal elements play a role in the dynamic regulation of the cytoskeleton that underlies cellular behavior. For example, HspB1 has been shown to be required for cellular migration in a variety of cell types: leukocytes, smooth muscle, neutrophils and fibroblasts [44]–[47]. Its role in migration is via regulation of actin filament polymerization at the leading edge of the cell, as well as in focal adhesions that connect the actin cytoskeleton to the extracellular matrix. In addition to influencing cell migration, HspB1 also plays a role in axonal growth from neurons [36], [37], [39], [48], [49]. We have suggested that the regulation of actin dynamics by HspB1 is important in axonal growth from primary neurons, and have previously demonstrated colocalization of HspB1 with F-actin and tubulin in lamellopodia, filopodia and focal contacts at the earliest stages of sensory neurite growth and in mature axons and growth cones [37], [39]. Using phosphorylation mutants of HspB1, we have recently reported that phosphorylation (or more specifically the oligomerization) status of HspB1 influences neurite growth and colocalization of HspB1 with F-actin in growth cones [36]. However the influence of phosphorylation was not clear cut as that observed in other cellular models. For example, HspB1-EE (fully phosphorylated and unable to be dephosphorylated) resulted in the lowest amount of total neurite growth, which could be consistent with decreased actin polymerization. In contrast, the non-phosphorylatable construct (HspB1-AA) showed similar growth to the WT HspB1 with a more patchy colocalization with F-actin, an effect that is difficult to explain based on the models that suggested that nonphosphorylated HspB1 inhibits F-actin polymerization via sequestration of actin monomers [21], or capping of the barbed ends of actin filaments [50]. Unlike experiments with purified HspB1 and actin, the situation in the cellular context is much more complex, with HspB1 being able to be rapidly phosphorylated and dephosphorylated in response to the changing cellular environment.

The current study was undertaken as part of our efforts to understand interactions of actin and HspB1 in a cellular context using a model system amenable to biochemical approaches. Here in the normal cells, we observed that the cellular distribution of HspB1, pHspB1 and F-actin in lamellopodia and at the leading edges of cells is consistent with a role in normal cellular attachment, spreading and cellular motility (Fig. 1). Specific isolation of F-actin from total cell lysates or the cytoskeletal fraction also precipitated HspB1. Non-phosphorylated HspB1 was observed in a complex with F-actin in the control cells, as was the relatively small fraction of phosphorylated HspB1. In this situation the association of HspB1 and F-actin likely plays a role in the normal adhesion and motility of these PC12 cells, with phosphorylated HspB1 potentially supporting actin polymerization needed for cellular adhesion and migration [47], [51].

### Role of HspB1 in Cytoskeletal Protection

Part of HspB1’s protective role in stressed cells has been attributed to its interactions with actin, resulting in increased actin filament stability. During various stresses HspB1 has been reported to increase the stability of the actin cytoskeleton effectively preventing actin fragmentation [52]–[56]. HspB1 has been reported to interact with actin as a barbed end actin capping protein [57], and its actions in protecting the actin filament cytoskeleton have been attributed to this actin capping ability [52]. However, it has also been suggested that HspB1 impairs actin filament polymerization by sequestering actin monomers rather than by capping [21]. Protection of the actin cytoskeleton by HspB1 appears to be dependent on the ability of HspB1 to bind to denatured actin filaments, preventing their aggregation and facilitating reformation [58]. In the actin-capping model, HspB1 was thought to cap actin filaments as a nonphosphorylated monomer with phosphorylation resulting in dissociation from the actin filament and elongation [23], [44], [50], [57], [59]. The actin sequestering model suggests that nonphosphorylated HspB1 binds actin monomers resulting in an increase in the G-actin pool and a subsequent decrease in actin filament levels; upon phosphorylation HspB1 dissociates from the actin monomers, which are then available for filament elongation [21]. A recent *in vitro* study provides support for a model with HspB1 binding along the lengths of actin filaments, but not acting as a capping protein [30].

HspB1 is known to interact directly with actin *in vitro* as described above, but whether this is also true in cells has been unclear. While a number of studies have shown co-localization of HspB1 with F-actin and redistribution of both after various stimuli using confocal microscopy, few have concomitantly studied whether there is a biochemical association with F-actin. Furthermore, the majority of these investigations have employed cells in which HspB1 had been overexpressed [23], [24], [27], [47], [60], [61]. Although there has been little evidence for a direct association of HspB1 and actin *in vivo*, recent studies have shown that actin exists in complexes of immunoprecipitated HspB1 [24], [62]. One of these demonstrated that actin could be interacting with HspB1 based on immunoprecipitation of overexpressed tagged fish HspB1 from mammalian cells [24]. In this study, the interaction between fHspB1 and actin after HS was observed to decrease by 3 hrs, but then increased by 24 hrs. In our study, we were primarily interested in the response of PC12 cells to a HS over the short term when phosphorylation of HspB1 increases rapidly; in PC12 cells in response to HS, pHsp is detectable as early as 5 min after a 15 min HS, with a maximum reached by 1 hr [41]. Unlike the former study, we observed association of HspB1 with F-actin in the control cells and an increased association of pHspB1 with F-actin immediately after the termination of the HS.

An elegant study by Jia and colleagues quite clearly demonstrates that endogenous HspB1 interacts not only with actin but also with several other cytoskeletal proteins and actin-binding proteins [62]. In our experiments, while we did not exhaustively investigate the presence of other proteins in the precipitated complexes, we did not detect the presence of tubulin, neurofilament, nestin or Daxx (data not shown), suggesting that interactions of HspB1 and the cytoskeleton may vary in different cells.

In our investigation, we performed pull-down and immunoprecipitation assays in order to determine any interaction between HspB1 and F-actin. Under all conditions HspB1 interacted with F-actin with seemingly high selectivity, based upon our approach of performing both direct precipitation of F-actin and the reverse approach of HspB1 IP. Our results show that HspB1 is found in a complex with F-actin both in non-phosphorylated and phosphorylated forms, with an increased association of pHspB1 with actin after stress. In the normal cells, the cellular distribution of HspB1, pHspB1 and F-actin is consistent with a role in cellular motility, being present in lamellopodia and at the leading edges of cells (Fig. 1). Upon HS, there was a relatively rapid (within 30 min) reorganization of the cytoskeleton accompanied by a redistibution and increased phosphorylation of HspB1 (Fig. 2). This is evident at the level of cellular localization (Figs 1–3) and also at the biochemical level (Figs 4, 6–8). Specific isolation of F-actin from total cell lysates or the cytoskeletal fraction also precipitated HspB1. Non-phosphorylated HspB1 was observed in a complex with F-actin in both the control and stressed cells. A relatively small fraction of HspB1 is phosphorylated in the control cells. In this situation according to the proposed models, phosphorylated HspB1 would support actin polymerization needed for cellular adhesion and migration [47], [51]. After HS there is an increased phosphorylation and redistribution of HspB1 to the cytoskeletal fraction. It is clear that a significant fraction of the HspB1 pulled down with F-actin is phosphorylated on both the S15 and S86 sites, and that this is attenuated by the presence of the p38 MAPK inhibitor, SB203580. The increase in phosphorylation and increased association with F-actin is consistent with the role of HspB1 in protecting actin filaments by inhibiting actin fragmentation [23], [53]. The reverse pull-down experiments provide similar results. Immunoprecipitation of HspB1 also brings down actin and, in the cytoskeletal pellet at least, it is highly likely that this is also F-actin. With stress there is an increased amount of HspB1 precipitated, and an apparent corresponding increase in the amount of actin, although ratio of actin:HspB1 does not differ between conditions (Fig. 7 and 8).

These results are the first that we are aware of to combine both cellular and molecular approaches to show cellular localization and a direct demonstration of an interaction between endogenous HspB1 and F-actin, using a method that specifically isolates endogenous F-actin. However, based upon the reports of others, we also are aware that despite our evidence that we cannot rule out some other indirect interactor that plays a role in linking the response of the cytoskeleton to HspB1.

### Conclusions

HspB1 is associated with the actin cytoskeleton in both control and heat-shocked cells. HspB1 phosphorylated on both the S15 and S86 sites is detectable by immunocytochemistry in normal cells and this is increased after HS, but attenuated by treatment with the p38 MAPK inihibitor. HspB1 is found distributed primarily throughout the cytoplasm in the normal cells and is redistributed after HS to the cytoskeletal fraction, where it is detected as colocalized with F-actin. HS results in the phosphorylation of HspB1, which also colocalizes with F-actin and is significantly increased in association with F-actin after stress. These results directly demonstrate that HspB1 interacts with F-actin and that both phosphorylated and non-phosphorylated HspB1 are found to be present in F-actin complexes.

## Supporting Information

Figure S1 **Densitometric analyses of relative gray levels of PC12 cell ICC demonstrating the significant increase in pSer15-HspB1 and pSer86-HspB1 with heat shock.** Relative densitometric quantification and statistical analyses of cells imaged by confocal microscopy analyzed using ImageJ. Values expressed are the average grey values for pSer15-HspB1, pSer86-HspB1 and total HspB1 (+/− S.E.M., n = 100). **: P<0.01; ***: P<0.001
(TIF)
