# Supplementary figures and images for: Cell Stress Promotes the Association of Phosphorylated HspB1 with F-Actin
Source: PLoS One. 2013 Jul 10;8(7):e68978. doi: 10.1371/journal.pone.0068978 (PMC3707891; doi:10.1371/journal.pone.0068978)

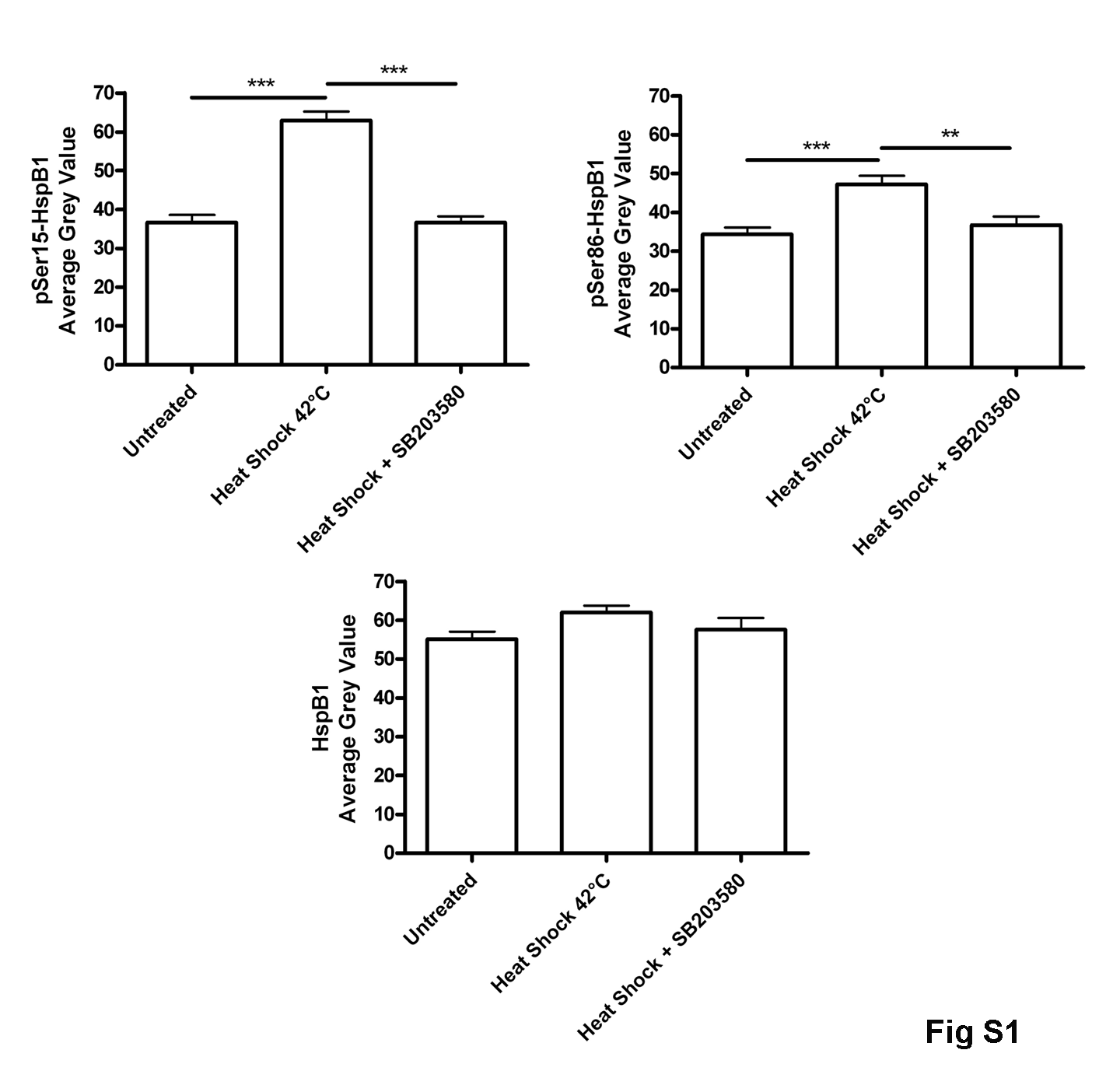

Supplement: Figure S1 — Densitometric analyses of relative gray levels of PC12 cell ICC demonstrating the significant increase in pSer15-HspB1 and pSer86-HspB1 with heat shock. Relative densitometric quantification and statistical analyses of cells imaged by confocal microscopy analyzed using ImageJ. Values expressed are the average grey values for pSer15-HspB1, pSer86-HspB1 and total HspB1 (+/− S.E.M., n = 100). **: P<0.01; ***: P<0.001 (TIF) [file pone.0068978.s001.tif]
